# Using Optic Nerve Sheath Diameter for Intracranial Pressure (ICP) Monitoring in Traumatic Brain Injury: A Scoping Review

**DOI:** 10.1007/s12028-023-01884-1

**Published:** 2023-12-19

**Authors:** Karol Martínez-Palacios, Sebastián Vásquez-García, Olubunmi A. Fariyike, Chiara Robba, Andrés M. Rubiano, the noninvasive ICP monitoring international consensus group, Fabio Silvio Taccone, Frank Rasulo, R Rafael Badenes, David Menon, A Aarti Sarwal, D Danilo Cardim, Marek Czosnyka, Mohammad Hirzallah, Thomas Geeraerts, Pierre Bouzat, Pier G. Lochner, Marcel Aries, Yu Lin Wong, Yasser Abulhassan, Gene Sung, Hemanshu Prabhakar, Gentle Shrestha, Luis Bustamante, Manuel Jibaja, Juan Pinedo, Diana Sanchez, Julio Mijangos Mendez, Franly Vásquez, Dhaval P. Shukla, Getaw Worku, Abenezer Tirsit, Bhagavatula Indiradevi, Hamisi Shabani, Amos Adeleye, Thangaraj Munusamy, Amelia Ain, Wellingson Paiva, Daniel Godoy, Sérgio Brasil, Chiara Robba, Andrés Rubiano, Sebastián Vásquez-García

**Affiliations:** 1 Neuroscience Institute, Universidad El Bosque, Bogotá, Colombia; 2 MEDITECH Foundation, Calle 7a #44-95, Cali, Colombia; 3 Universidad del Rosario, Bogotá, Colombia; 4 Stanford University School of Medicine, Palo Alto, CA USA; 5 Department of Anesthesia and Intensive Care, Policlinico San Martino, Genoa, Italy

**Keywords:** Intracranial pressure (ICP), Monitoring, Traumatic brain injury (TBI), Intracranial hypertension, Noninvasive monitoring, Invasive monitoring, Optic nerve sheath diameter, Optic nerve ultrasound

## Abstract

**Introduction:**

Neuromonitoring represents a cornerstone in the comprehensive management of patients with traumatic brain injury (TBI), allowing for early detection of complications such as increased intracranial pressure (ICP) [1]. This has led to a search for noninvasive modalities that are reliable and deployable at bedside. Among these, ultrasonographic optic nerve sheath diameter (ONSD) measurement is a strong contender, estimating ICP by quantifying the distension of the optic nerve at higher ICP values. Thus, this scoping review seeks to describe the existing evidence for the use of ONSD in estimating ICP in adult TBI patients as compared to gold-standard invasive methods.

**Materials and Methods:**

This review was conducted in accordance with the Joanna Briggs Institute methodology for scoping reviews, with a main search of PubMed and EMBASE. The search was limited to studies of adult patients with TBI published in any language between 2012 and 2022. Sixteen studies were included for analysis, with all studies conducted in high-income countries.

**Results:**

All of the studies reviewed measured ONSD using the same probe frequency. In most studies, the marker position for ONSD measurement was initially 3 mm behind the globe, retina, or papilla. A few studies utilized additional parameters such as the ONSD/ETD (eyeball transverse diameter) ratio or ODE (optic disc elevation), which also exhibit high sensitivity and reliability.

**Conclusion:**

Overall, ONSD exhibits great test accuracy and has a strong, almost linear correlation with invasive methods. Thus, ONSD should be considered one of the most effective noninvasive techniques for ICP estimation in TBI patients.

**Supplementary Information:**

The online version contains supplementary material available at 10.1007/s12028-023-01884-1.

## Introduction

Neuromonitoring is essential for the management of patients with traumatic brain injury (TBI), allowing early detection of potential insults such as increased intracranial pressure (ICP), which may precipitate a cascade of events (from ischemia to brain herniation) that warrant proper and timely treatment [1–3]. Thus, efficient, reliable, and widely available tools for such ICP monitoring are also equally essential. Currently, the gold standard for neuromonitoring consists of intraventricular and intraparenchymal transducers; however, these techniques are costly and require skilled personnel. As a consequence, these techniques are usually restricted to high-level centers [4, 5]. Invasive devices are also generally contraindicated in patients with bleeding disorders while also carrying the risk of infection and malfunction [6–9]. This has led to a search for noninvasive modalities for ICP monitoring that are inexpensive, reliable, reproducible, and tailored for point-of-care applications [10, 11]. Different methods are described in the literature for noninvasive ICP (nICP) estimation, which include, but are not limited to, optic nerve sheath diameter (ONSD) measurement as assessed by ultrasound, computerized tomography or magnetic resonance imaging [12, 13], transcranial Doppler-derived indices (e.g., pulsatility index and flow velocities) [14], and the measurement of pupil size and other dynamic pupillary variables (Neurologic Pupillary Index, NPi, latency, constriction velocity, and dilation velocity) [15]. The goal of this work is to characterize the evidence concerning exclusively ONSD abilities to estimate nICP in adult patients with TBI.

## Review Questions

The objective of this scoping review is to describe the extent and type of evidence for nICP monitoring in TBI using ONSD, as compared with standard invasive methods in the adult population. Applying the Patient, Concept, Context (PCC) framework, the following specific questions were formulated:

1. Which methods are available for nICP monitoring using ultrasound-measured ONSD?
2. What evidence exists for the association and accuracy of nICP monitoring using ultrasound-measured ONSD versus invasive monitoring for ICP estimation?

## Methods

This scoping review was conducted in accordance with the Joanna Briggs Institute methodology for scoping reviews.

### Inclusion Criteria

#### Participants

This scoping review considered studies including patients over 18 years old suffering from TBI who underwent nICP monitoring using ONSD and required diagnostic invasive ICP (ICPi) monitoring for ICP estimation. All studies in the pediatric population (defined here as < 18 years) were excluded.

#### Concept

The concept of this scoping review was to include studies that investigated nICP monitoring by ONSD in adult patients with all degrees of TBI (mild, moderate, and severe) as compared with the analysis derived from gold-standard invasive methods. Topics in this concept include, but are not limited to, device features, methodological details, variables derived from said methods, the diagnostic accuracy of each method in detecting intracranial hypertension, the reliability of these methods, and the sensitivity and specificity of a specific ONSD method in the diagnosis of intracranial hypertension.

#### Context

This scoping review did not consider the specific race, gender, or geographic location of participants in the selected studies. Given that the anatomy and pathophysiology of TBI within the pediatric population differ substantially from those of their adult counterparts, especially in the mid and lower age ranges (e.g., < 14 years and > 50 years), and because of the large amount of literature that consider “adults” by a cutoff of 18 years, exclusion was determined solely by participant age, with only studies conducted in adults > 18 years being included.

#### Types of Sources

The present scoping review assessed both experimental and quasi-experimental study designs including randomized controlled trials, nonrandomized controlled trials, before-and-after studies, and interrupted time-series studies. In addition, analytical observational studies including prospective and retrospective cohort studies, case–control studies, and analytical cross-sectional studies were considered for inclusion. This review also considered descriptive observational study designs including case series, individual case reports, and descriptive cross-sectional studies for inclusion. Qualitative studies that focus on qualitative data were also considered, including but not limited to, designs such as phenomenology, grounded theory, ethnography, qualitative description, action research, and feminist research. In addition, systematic reviews that met the inclusion criteria were also considered, depending on the research question.

### Search Strategy

An initial search in EMBASE and PubMed was undertaken, aimed at locating published studies in the adult population between January 2012 and June 2022 so as to obtain the most updated evidence and technological advances on the subject and because, specifically from 2012, the results by year in the two employed databases (exhibited in bar charts in their websites) showed an important increase in the publication of studies in the topic. Additionally, studies published in any language were included, as the available and useful literature was in a variety of languages. Studies that contained noninvasive monitoring with techniques other than ONSD were excluded. Studies containing ICPi or nICP monitoring for the diagnosis of intracranial hypertension from etiologies other than TBI were also excluded. We also exclude narrative reviews in the topic because, although in general they are comprehensive in the information that they provide, they are influenced subjectively by the authors and may have outdated sources. A detailed search strategy for both databases is contained in online Appendix 1.

### Source of Evidence Screening/Selection

**Fig. 1:**
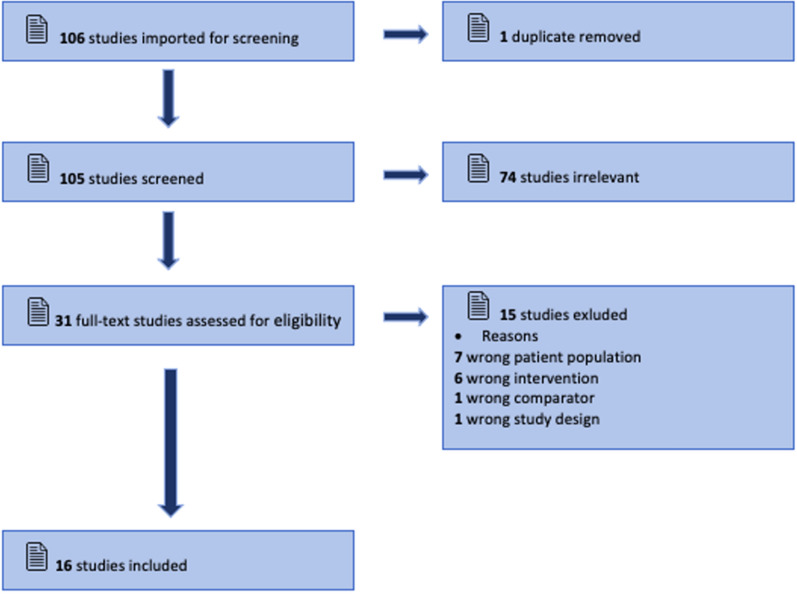
Extraction methodology

The initial EMBASE and PubMed search yielded 106 studies. All identified citations were collated and uploaded into Covidence, and one duplicated study was removed. Studies were screened by two independent researchers (KM and SV) and one collaborator (OF). After examining 105 titles and abstracts for inclusion, 74 irrelevant studies were removed, 31 full-text studies were assessed for eligibility, and 15 studies were excluded for reasons described in Fig. 1. The results of the search are reported using the Preferred Reporting Items for Systematic Reviews and Meta-Analyses extension for Scoping Reviews checklist [16].

## Results

**Fig. 2:**
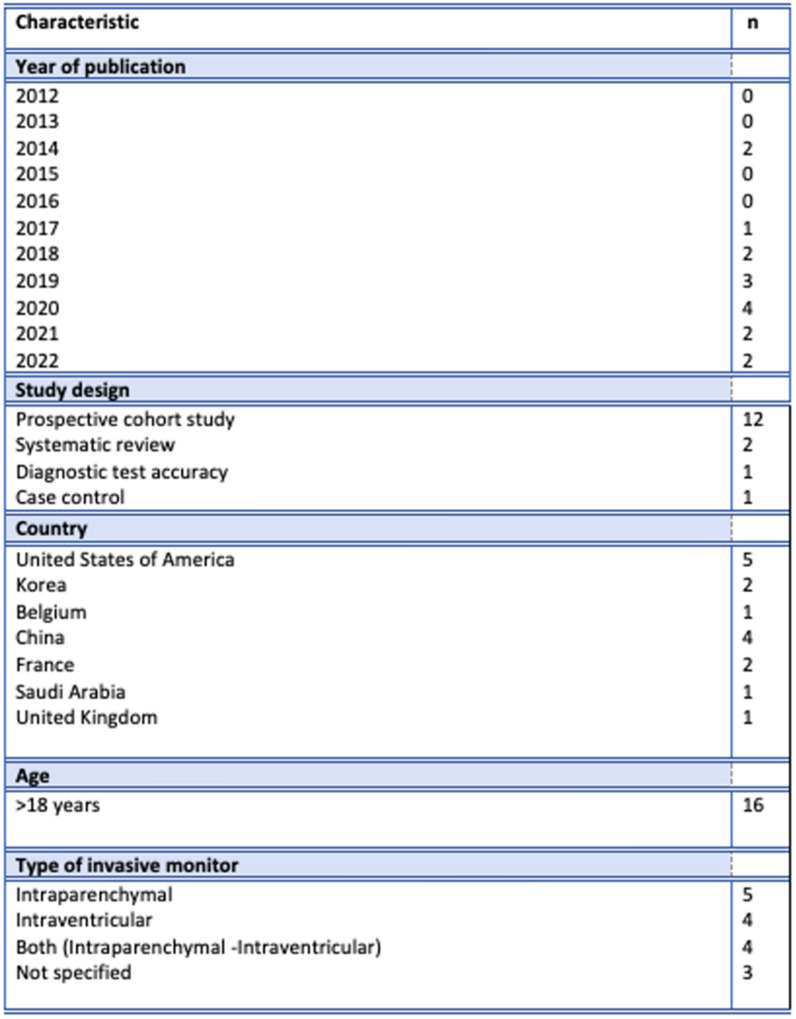
Characteristics of included publications

After reviewing and applying inclusion and exclusion criteria, 16 studies were included for final analysis. We did not find any qualitative study with designs such as phenomenology, grounded theory, ethnography, qualitative description, action research, and feminist research. Figure 2 provides the characteristics of the included publications. Table 1 provides the extracted information based upon the formulated research questions.

**Table 1:** ONSD-based methods for ICP estimation and their correlation with ICPi

First author, year of publication	Country	Type of study	Number of participants	Technique/method of nICP estimation	Method of invasive monitoring	Cutoff ONSD value for an ICP ≥ 20 mm Hg	nICP vs. ICP monitoring	Conclusions
Lee [28]	Korea	Systematic review	4 studies	All studies measured ONSD at 3 mm behind the globe/optic disk/papilla All studies used the average ONSD (ONSD of right eye + ONSD of left eye/2) for evaluating the diagnostic feasibility of measuring the ONSD	Not specified	Not specified	- The sensitivity and specificity were 0.91 and 0.82, respectively for sonographic ONSD in the prediction of intracranial hypertension	ONSD measurement may be a useful method for predicting raised ICP in adult patients with severe TBI
Singer [30]	United States	Cohort study	135 (66 TBI)	7.5 mHz ultrasound probe, 3 mm behind the globe for ONSD measurement	Not specified	Not specified	Average (right + left eye) correlation coefficient and *p* value: 0.498 and 0.292, respectively	ONSD in isolation shows a moderate correlation with invasive ICP
Robba [23]	Belgium	Cohort study	100 (30 TBI)	7.5-MHz linear ultrasound probe, with the patient in the supine position and with head elevated to 30° Probe oriented perpendicularly in the vertical plane and at around 30° in the horizontal plane on the closed eyelids of both eyes Measurements were made in the axial and sagittal planes, 3 mm behind the retina in both eyes, and the final ONSD value was calculated by averaging four measured values Abnormal ONSD was considered to be ≥ 6 mm	Intraventricular and Intraparenchymal	5.3 mm	In the TBI subgroup: There was a significant, moderate correlation between invasive ICP and ONSD (r = 0.53, *p* = 0.002) The AUC to predict intracranial hypertension was 0.78 [95% CI: 0.62–0.95] for ONSD A ONSD > 5.3 mm had 67% sensitivity and 73% specificity to predict intracranial hypertension (defined as ICP > 20 mm Hg)	The noninvasive ICP estimated by ONSD method was significantly correlated with invasive ICP; however, the correlation was moderate-to-weak
Al-Mufti [31]	United States	Systematic review	100 studies	Ultrasound-measured ONSD (US_ONSD)	Intraventricular and Intraparenchymal	6 mm	US_ONSD measurements are accurate for assessing ICP, with sensitivity and specificity approaching 90% and 85%, respectively US_ONSD < 5 mm could be considered as “normal ICP,” while measurements > 6 mm represent an elevated ICP (ICP > 20 mm Hg)	US_ONSD measurements seem to be accurate for estimating elevated ICP. The cutoffs given represent data extracted from the studies analyzed in this systematic review
Martin [33]	France	Cohort study	54	ONSD: 7.5-MHz probe, 3 mm behind the globe	Intraparenchymal	5.6 mm	ICP of > 20 mm Hg for at least 5 min ONSD: AUC ROC = 0.73 (95% CI 0.59–0.86) Sensitivity and NPV of 100% for a ONSD cutoff of 5.6 mm	ONSD, with the cutoff given, showed a good capability to detect ICP > 20 mm Hg for at least 5 min
Youm [19]	South Korea	Cohort study	50	7 to 12 MHz linear-array transducer Mean value from 3 ultrasound images from the bilateral eyes was recorded and was obtained 3 mm from the retina The ONSD was measured using 2 methods: the ONSDI, measured as the distance between the external borders of the hypoechogenic leptomeninges (dura mater), and the ONSD including the subarachnoid space, defined as the subarachnoid diameter (ONSDE) The ETD for the ONSD/ETD ratio was measured from the inner margin of one eyeball to that of the other. The mean value of the largest diameters in the bilateral eyeballs was calculated through initial brain CT (important caveat)	Intraventricular	6.15 mm	The ONSDE, ONSDI, and ONSD/ETD ratio were significantly associated with ICP (*p* = 0.005, *p* < 0.001, and *p* < 0.001, respectively) A close association of the ONSDI and ONSD/ETD ratio with the ICP was demonstrated (R = 0.511 and 0.59) The cutoff values (correlated with invasive ICP greater than or equal to 20 mmHg) of the ONSD/ETD ratio, ONSDI, and ONSDE were 0.264, 6.15, and 5.05, respectively	The ONSD/ETD ratio is the best predictor of increased ICP among the three methods, with the second being ONSDI
Frumin [17]	United States	Cohort study	27	With a 5–10 MHz linear probe, ONSD was measured from outer wall to outer wall 3 mm posterior to the globe Binocular mean ONSD was taken as the final measurement	Intraventricular	5.2 mm	The ONSD cutoff value of ≥ 5.2 mm accurately predicted ICP > 20 mm Hg with a sensitivity of 83.3% (95% CI 35.9–99.6%) and specificity of 100% (95% CI 83.9–100%) The positive predictive value of ONSD ≥ 5.2 mm for invasive ICP was 100% (95% CI = 48–100%) and the negative predictive value of ONSD less than 5.2 mm was 95.5% (95% CI = 77.2–99.9%)	ONSD cutoff values showed a good correlation with invasive ICP values > 20 mm Hg
Wang [21]	China	Cohort study	20	An axial cross-sectional image of the optic nerve was obtained. The optic nerve sheath diameter was measured 3 mm behind the retina. Each eye was measured 3 times, and the average value of 6 measurements was recorded	Not specified	5.55 mm	Admission ONSD of > 5.55 mm was shown to have a sensitivity of 81.8% and a specificity of 88.9% for prediction of intracranial pressure increase (AUC = 0:919; 95%CI 0:798–1.0; *p* < 0.002) with a median ICP of 22.75 mmHg No head-to-head value comparison between ONSD in mm vs. ICP in mm Hg	ONSD using the described cutoff can be useful for the noninvasive detection of elevated ICP
Soliman [20]	Saudi Arabia	Cohort study	40	Measurements taken 3–4 mm behind the vitreoretinal interface, in perpendicular direction to the axis of the nerve ONSD was recorded for both eyes, while the mean value of the two measurements was used as the final value in mm More information on sonographic quality criteria (Table 1 original article)	Intraparenchymal	6.4 mm 6.6 mm	ONSD measurements were strongly correlated to invasively monitored ICP values (*r* = 0.74, *p* < 0.0001) Correlation values: ICP of 20 mmHg: ONSD cutoff value of 6.4 mm, Sensitivity: 85.3%, Specificity: 82.6% ICP of 25 mmHg: ONSD cutoff value of 6.6 mm, Sensitivity: 87.2%, Specificity: 80.2%	When applying the described sonographic quality criteria, ONSD is strongly correlated to ICP in severe TBI
Kashyap [22]	United States	Cohort study	11	ONSD measured in axial plane and 3 mm behind the globe	Intraventricular	Not applicable	The mean ONSD pretreatment was 6.24 mm (± 1.28 mm), and the mean ONSD post treatment was 5.62 mm (± 1.22 mm), resulting in a mean decrease of 0.62 mm (*P* = 0.0001) The mean pretreatment ICP was 13.48 mmHg (4.53 mm Hg) and the mean post treatment ICP was 10.14 mm Hg (3.45 mm Hg), yielding a mean decrease in ICP of 3.33 mm Hg (*P* = 0.0001)	ONSD exhibited a positive linear correlation with invasive ICP
Zhou [24]	China	Cohort study	73	7.5 MHz linear probes Probe oriented 30º above parallel on both closed eyes with the head elevated to 30º in the supine position at approximately 30º on the length of the plane and at the horizontal surface Measurements of the axial and sagittal planes of both eyes were made, such that the visible widest diameter behind the retina was 2.8 mm	Intraventricular and Intraparenchymal	Not applicable	ICP vs. ONSD data were presented only describing maximum, minimum and mean (SD) values, *p* values, and corresponding correlation coefficients®: ICP (mm Hg): Minimum: 5, maximum: 17, mean ± SD: 10.24 ± 3.51 Optic nerve sheath diameter (mm): Minimum 4, maximum 6, mean ± SD 4.77 ± 0.43, r = 0.8717, *p* < 0.0001	Among the methods studied, ONSD displayed the best correlation with invasive ICP measurements
Launey [29]	France	Cohort study	93	3 mm longitudinally from the location of the retina in each eye and an axis perpendicular to the optic nerve. The mean value of the right and left ONSD was used as the final measure	Intraventricular and Intraparenchymal	5.8 mm	There was a significant correlation between ICP and ONSD measurements before and after mannitol infusion (*r*^2^ = 0.54; *p* < 0.002) Value correlations: ICP 20 mm Hg with an ONSD of 5.8 mm ICP 25 mm Hg with an ONSD of 6.1 mm ICP 30 mm Hg with an OSND of 6.4 mm Key point: A threshold of 5.8 mm for ONSD was proposed to distinguish ICP of more than 20 mm Hg	ONSD is a useful, user-friendly, and noninvasive tool to detect ICP elevation. It is well correlated with ICP. Under dynamic conditions, this correlation remains valid, even after osmotherapy with mannitol
Robba [32]	United Kingdom	Cohort study	64 (45 TBI)	7.5-MHz linear ultrasound probe The probe was oriented perpendicularly in the vertical plane and at around 30° in the horizontal plane on the closed eyelids of both eyes of individuals in supine position with head elevated to 30° Axial and sagittal planes of the widest diameter visible 3 mm behind the retina in both eyes were used ONSD value was calculated by averaging 4 measured values	Intraparenchymal	5.85 mm	The correlation coefficient between the ONSD method and ICP averaged per patient was *R* = 0.76 ONSD had the best AUC for discriminating cases with intracranial hypertension (ICP 20 mm Hg) from cases without it (AUC = 0.91, 95%CI 0.88 ± 0.95) The best ONSD cutoff values for prediction of intracranial hypertension were 5.85 mm The formula of the derived models that best fitted the data was: nICP_ONSD = 5.00 × ONSD—13.92 (mm Hg)	ONSD showed a good correlation with invasive ICP values, being more in terms of the cutoff given, but also with the aforementioned formula of derived models
Du [26]	China	Cohort study	52	Ultrasound-ONSD/ETD ratio and ultrasound-ONSD: The maximum external diameter of ONSD (ultrasound-ONSD) at 3 mm behind the ball and the maximum diameter of ETD (parallel lens) on this plane were measured Both eyes were measured three times, and the values were averaged as the final ONSD and ETD	Intraventricular	5.53 mm	Accuracy comparison of ultrasound-ONSD/ETD ratio, ultrasound-ONSD: The AUC of ROC curve was 0.920 (95% CI: 0.877–0.964), 0.870 (95% CI 0.798–0.910) The corresponding threshold (for ICP > 20 mm Hg) values for ONSD/ETD ratio and ultrasound-ONSD were 0.25 (sensitivity of 90%, specificity of 82.3%) and 5.53 mm (sensitivity of 80%, specificity of 79.3%) The predictive effect of ultrasound-ONSD/ETD, ultrasound-ONSD on intracranial hypertension was all consistent with that of invasive ICP (Kappa = 0.710, *P* < 0.05; Kappa = 0.602, *P* < 0.05)	ONSD/ETD ratio outperformed ONSD both in terms of correlation with ICP when values were > 20 mm Hg and in terms of predicting intracranial hypertension
Wang [27]	China	Case control study	35 TBI, 40 healthy controls	US_ONSD measurements were performed in patients in the semisupine position with the head raised by 20°–30° ONSD was assessed 3 mm behind the globe, and the diameter was measured perpendicularly to the optic nerve sheath The recorded ONSD was the mean value of 3 measurements, and both the left and right ONSDs were recorded	Intraparenchymal	5.83 mm (**for ICP ≥ 22 mm Hg)	A significant linear correlation was found between ONSD and ICP (*r* = 0.771, *p* < 0.0001) The best ONSD cutoff value for detecting ICP above 13 mm Hg was 5.48 mm, yielding sensitivity and specificity of 91.1% and 88.0%. The negative and positive predictive values were 78.57% and 91.11%, respectively The best ONSD cutoff value for detecting elevated ICP (above 22 mm Hg) was 5.83 mm, with sensitivity and specificity of 94.4% and 81.0%. Negative and positive predictive values were 97.67% and 62.96%	Ultrasonographic ONSD is strongly correlated with invasive ICP measurements and may serve as a sensitive and noninvasive method for detecting elevated ICP in TBI before and after decompressive craniectomy
Agrawal [18]	United States	Diagnostic test accuracy study	120	ONSD was measured in the axial plane 3 mm behind the retina, with a 13–6 MHz linear-array probe with orbital imaging settings and a high-resolution optimization setting Optic disk elevation was measured as the maximum height of the optic disk above the retina in mm	Intraparenchymal	7.2 mm (**for ICP ≥ 22 mm Hg)	The AUC of ONSD for ICP greater than 25 mm Hg was 0.76 (0.67–0.83; AUC = 0.5; *p* = 0.0395). The optimal threshold was greater than 0.72 cm, with sensitivity 83% (95% CI 36–100%) and specificity 76% (95% CI 67–84%) The AUC of ONSD for ICP greater than 22 mm Hg was 0.81 (0.73–0.87; AUC = 0.5; *p* < 0.0001). The optimal threshold was greater than 0.72 cm, with sensitivity 82% (95% CI 48–98%) and specificity 79% (95% CI 70–86%) The AUC of ODE for ICP greater than 22 mm Hg was 0.84 (0.76–0.90; AUC = 0.5; *p* < 0.0001). Optimal ODE threshold was greater than 0.04 cm, with sensitivity 90% (95% CI 56–100%) and specificity 71% (95% CI 61–79%) ONSD was associated with continuous ICP measurements (*p* = 0.0002) and also predictive of ICP dichotomized at less than or equal to 22 mm Hg versus greater than 22 mm Hg. The OR was 3.72 (1.68–8.24; *p* = 0.0014) for every 0.1 unit increase in ONSD, with AUC 0.88	Both ONSD and ODE (although slightly greater for ONSD) showed good correlations with invasive ICP values ≥ 22 mm Hg

*ABP* arterial blood pressure (in those studies, mean arterial pressure), *AUC* area under curve, *CI* confidence interval, *ETD* eyeball transverse diameter, *ICP* intracranial pressure, *ICPi* invasive ICP, *nICP* noninvasive intracranial pressure, *ODE* optic disc elevation, *ONSD* optic nerve sheath diameter, *ONSDE* subarachnoid diameter, *ONSDI* dura mater diameter, *OR* odds ratio, *ROC* receiver operating characteristic curve, *US_ONSD* ultrasound-measured ONSD

### Methods for nICP Monitoring Using Ultrasound-Measured ONSD

After reviewing in detail each of the studies included, the reported technical settings were identical in terms of probe frequency (a 7.5 MHz linear probe), with the exception of three studies that mentioned a range of frequencies rather than any single one [17–19].

Regarding the position of the ONSD measurement, all studies reported an initial measurement 3 mm behind the globe, retina, or papilla, whereas one study measured 2.8 mm behind the globe, and another in a range of 3–4 mm [20, 21]. Probe orientation for ONSD ultrasound was mentioned in four studies (Table 1). In three studies, the orientation was axial only [1, 21, 22]. In one, orientation was both axial and sagittal [23]. In the previous study and one other, a description of patient positioning during the ultrasound study (head of bed at 30°) was made [23, 24].

Regarding the method of estimating the final ONSD measurement, ten studies reported how they obtained the final value in mm (Table 1). Some considered an average of four values (one axial and one sagittal measurement for each eye) [23, 25] while some used an average of three values [19, 26, 27] or an average of two values [17, 20, 21, 28, 29]. The remaining included studies did not mention any details regarding the methods for obtaining the final ONSD measurement [18, 22, 24, 30–32].

Besides isolated ONSD measurements for ICP estimation of correlation with ICPi, three studies assessed parameters apart from ONSD that leveraged ultrasonography such as eyeball transverse diameter (ETD) [26], ONSD/ETD ratio [19], and optic disk elevation (ODE) (estimated by measuring the maximum height of the optic disk above the retina) [3]. One study even went as far as to propose a formula-based nICP estimation based on ONSD values as follows [25]:

$${\mathrm{nICP}}_{\mathrm{ONSD}}=\left(5.00\cdot \mathrm{ONSD}\right)-13.92 \mathrm{mm} \mathrm{Hg}$$

Uniquely, one of these studies assessed three different ONSD measurements: the proper optic nerve (ON) diameter (between the pia mater), the measured distance between the external borders of the hypoechogenic leptomeninges or dura mater (ONSDI), and the ONSD including the subarachnoid space (SAS), defined as the subarachnoid diameter (ONSDE) [19]. Each method description along with its associated variables is summarized in Table 1.

### nICP by ONSD Versus ICPi Monitoring for ICP Estimation

Of the studies analyzed, only five did not mention a calculated correlation between ONSD and ICPi [21, 22, 24, 28, 30]. All studies except for two considered “elevated intracranial pressure” as greater than or equal to 20 mm Hg [18, 27]. Two studies specifically used the term “intracranial hypertension” to refer to values above this threshold (Table 1) [23, 25]. On the other hand, four studies assessed different ICP thresholds and estimated their corresponding ONSD threshold values (Table 1): Launey et al. reported ICPi values of 20 mm Hg, 25 mm Hg, and 30 mm Hg, Soliman et al. reported values of 20 mm Hg and 25 mm Hg, Wang et al. of 13 mm Hg and 22 mm Hg, and Agrawal et al. of 22 mm Hg and 25 mm Hg [18, 20, 27, 29].

Regarding the ICPi monitoring technique, the vast majority of studies considered either intraparenchymal techniques, intraventricular techniques, or both, and only three studies did not mention the type of ICPi method employed (Table 1) [21, 28, 30]. None of the analyzed studies made any specifications about the side of injury or type of injury (e.g., contusion vs. subdural hematoma) with respect to ICPi and nICP estimation or about the site of ICP probe placement in relation to the side of ONSD measurement. All the studies were performed in high-income countries (Fig. 2).

In a prospective cohort study of 27 patients with TBI, ONSD accurately predicted ICPi greater than 20 mm Hg with a sensitivity of 83.3% (95% Confidence Interval CI [35.9%, 99.6%]) and a specificity of 100% (95% CI [83.9%, 100%]). The positive predictive value of an ONSD value greater than or equal to 5.2 mm was 100% (95% CI [48%,100%]), and the negative predictive value of ONSD less than 5.2 mm was 95.5% (95% CI [77.2%, 99.9%]) (Table 1). The receiver operating characteristic curve demonstrated an area under the curve of 0.865 [17].

In a diagnostic test accuracy study performed in 120 patients with TBI, both ONSD and ODE were analyzed. The optimal ONSD threshold for detecting ICPi greater than 25 mm Hg was 7.2 mm, with a sensitivity of 83% (95% CI [36, 100%]) and a specificity of 76% (95% CI [67%, 84%]). Notably, a cutoff of 7.2 mm similarly detected ICPi greater than 22 mm Hg and less than 25 mm Hg at the expense of a slight decrease in sensitivity and increase in specificity to 82% (95% CI [48%, 98%]) and 79% (95% CI [70%, 86%]), respectively. Meanwhile, ODE showed an optimal threshold of greater than 0.04 cm to detect an ICPi greater than 22 mm Hg, with a sensitivity of 90% (95% CI [56%, 100%]) and a specificity of 71% (95% CI [61%, 79%]) (Table 1) [18].

A prospective cohort study in 50 patients calculated variables apart from ONSD including ONSDI, ONSDE, and ETD for the ONSD/ETD ratio (Table 1). The values for ONSDE, ONSDI, and ONSD/ETD ratio were significantly associated with ICP (*p* = 0.005, *p* < 0.001, and *p* < 0.001, respectively). The greatest association with ICPi was with ONSD (*r* = 0.511) and ONSD/ETD ratio (*r* = 0.59) The cutoff values in terms of ONSD/ETD, ONSDI, and ONSDE for an ICPi of greater than or equal to 20 mm Hg were 0.264, 6.15, and 5.05, respectively [19].

A cohort study performed in 73 patients that correlated maximum, minimum, and standard deviation values for both ONSD and ICPi found a good correlation between the two modalities (*r* = 0.8717, *p* < 0.0001) in the setting of TBI [24].

A prospective cohort study performed in 40 patients using two ICPi cutoff values (20 and 25 mm Hg), found a strong correlation between ONSD values and invasive monitoring (*r* = 0.74, *p* < 0.0001), with an ONSD cutoff value of 6.4 mm for an ICPi of 20 mm Hg and of 6.6 mm for an ICPi of 25 mm Hg. Sensitivity and specificity were both more than 80% (Table 1) [20].

In a prospective cohort study of 20 patients with TBI, an ONSD greater than 5.55 mm was shown to have a sensitivity of 81.8% and a specificity of 88.9% for prediction of ICP increases (Area Under Curve 0.919; 95% CI [0.798,1.0], *p* < 0.002). The median ICPi value was 22.75 mm Hg, with no head-to-head value comparison between ONSD in mm versus ICP in mm Hg [21].

In another cohort study in 11 patients with TBI, a linear relationship between ONSD and ICPi was documented when a specific ICP-lowering treatment was applied, with an average posttreatment decrease in ONSD of 0.62 mm (*p* = 0.0001) and an average posttreatment decrease in ICPi of 3.33 mm Hg (*p* = 0.0001). Correlations were similar in the pretreatment stage (Table 1) [22].

Another prospective cohort study in 100 patients (30 with TBI) found a significant, moderate correlation between ICPi and ONSD (*r* = 0.53, *p* 0.002), with an ONSD cutoff value greater than 5.3 mm predictive of intracranial hypertension (ICPi > 20 mm Hg). This cutoff value was associated with a sensitivity of 67% and a specificity of 73% [23].

In a cohort study assessing ONSD and another ultrasound-estimated measurement, the ETD, in 52 patients, better accuracy was observed for the ONSD/ETD ratio over ONSD in isolation. An ONSD/ETD cutoff value was set at 0.25 (sensitivity of 90%, specificity of 82.3%), and an ONSD cutoff value was set at 5.53 mm (sensitivity of 80%, specificity of 79.3%) [26].

A Chinese case–control study with a total sample size of 75 (35 patients with TBI, 40 healthy controls), showed a significant correlation between ONSD and ICPi (*r* = 0.771, *p* < 0.0001). Here, the cutoff value for detecting ICP above 13 mm Hg was defined as 5.48 mm, with sensitivity and specificity of 91.1% and 88.0%, respectively. Similarly, the cutoff value for detecting ICP above 22 mm Hg was 5.83 mm, with sensitivity and specificity of 94.4% and 81.0%, respectively (Table 1) [27].

In a systematic review of four studies in patients with TBI, the sensitivity and specificity were 91% and 82% for sonographic ONSD in the prediction of intracranial hypertension [28].

A prospective cohort study in 135 patients (66 with TBI) in the United States described both a weak-moderate correlation (*r* = 0.498, *p* = 0.292) between ONSD and ICPi values. However, no data regarding other specific values nor a ICPi estimation method were specified [30].

A systematic review of 12 studies concluded that ultrasound ONSD measurements less than 5 mm could be considered “normal ICP,” whereas measurements greater than 6 mm represented an elevated ICP (ICP > 20 mm Hg). This ICP cutoff was considered accurate for assessing “ICP crisis,” with sensitivity and specificity approaching 90% and 85%, respectively [31].

Lastly, in a French study of 54 patients with TBI, an ONSD cutoff value of 5.6 mm had a sensitivity and negative predictive value of 100% for detecting ICPi greater than 20 mm Hg, with an area under the curve of 0.73 (95% CI [0.59–0.86]) [33].

## Discussion

According to the information extracted from the reviewed studies and their reported results, ONSD seems to have a great accuracy and good correlation with ICPi, both in isolated absolute values and in incremental or decremental changes. The relationship between ONSD and ICPi values is almost linear. Nevertheless, OSND is more useful in detecting high ICP than in identifying normal ICP, which is clinically useful given that undetected ICP elevation often leads to detrimental consequences in patients with TBI, whereas low or normal ICP is usually less concerning.

The theoretical principles of ONSD ultrasound measurements as an indirect estimation of ICP depend on an extensive number of pathophysiological phenomena, which are mainly related to cerebrospinal fluid (CSF) dynamics and ICP transmission to the space surrounding the ON [34]. Strictly speaking, the ON is not a nerve by histological terms, but instead a central nervous system (CNS) white matter tract that extends into the orbit, where it is surrounded by CSF throughout its entire length. Given this particular anatomical configuration, the ON is sensitive to ICP changes in its surrounding layers. The dura mater and, to a lesser extent, the arachnoid, allow the circulation and storage of CSF due to their ability to physically expand [35]. There are several theories regarding CSF dynamics as a whole that, in specific CNS areas such as the ON, take into account the role of other parameters in CSF dynamics, such as physics per se [36, 37], bridging veins and sagittal sinus pressures [38], and the rate of CSF production and drainage (via arachnoid villi and the glymphatic system) [34, 37], among others. However, there is no single unified theory that comprises and integrates all of these phenomena, and some deserve to be mentioned specifically with respect to the ON and ONSD.

Free, bidirectional communication between the intracranial SAS and the ON SAS (optic nerve subarachnoidal space) has been proposed in an attempt to define how the cranial CSF that enters the SAS of the ON could change its direction of flow against the volume gradient that directs it from the higher volume site of production (in the intracranial space) toward the SAS of the ON [35]. Two outflow routes have been proposed. The first is from the SAS of the distal portion of the ON, and the second via the glymphatic system [34, 35]. Thus, for now, we can speculate that a certain amount of CSF is already circulating around the ON in the SAS at baseline and that “extra fluid” (in the setting of TBI and other CNS diseases) in fact represents CSF redistribution as a compensatory mechanism in early-stage and mid-stage of ICP increases. This excess fluid occupies the SAS and leads to ONSD distention detected by ultrasound. Other possibly related mechanisms, such as impaired exchange between intracranial CSF and ON SAS, described in other diseases have not yet been considered in TBI, given the different disease processes and underlying pathophysiology [39]. As such, ONSD estimation indeed represents a noninvasive way of detecting ICP changes by assessing, from a CSF-dynamics point-of-view, early “buffering mechanisms” in cases of intracranial compliance compromise [40].

With respect to the sonographic techniques for ONSD evaluation and assessment of other similar parameters described in the analyzed studies, the information extracted was generally highly variable in terms of the probe’s plane of orientation/insonation [25, 41], patient positioning [23, 24], anatomical landmarks for ONSD measurement [19], and final ONSD measurement calculation methods (Table 1). It is worth mentioning that there were three additional proposed methods apart from ONSD for nICP estimation: ODE [18], ONSD/ETD ratio [19], and a formula-based method leveraging calculated ONSD values (Table 1) [25]. Some of these methods demonstrated better correlation with ICPi and even greater sensitivity and specificity than ONSD alone [18, 19]. Such methods may represent an attempt to individualize ONSD measurements by taking into account eyeball size and its relation to ONSD for each patient [19]; however, there are also recently published data that contrarily report no correlation between global size and ONSD in a healthy Latin American population, creating possible uncertainties regarding the usefulness of these techniques for nICP assessment [42]. On the other hand, these methods may constitute a sonographic assessment of optic disk changes, which may be detected earlier than changes in routine fundoscopy, specifically papilledema [18, 43]. Thus, specifically, the formula-based method proposed by Robba et al. may have a great potential for nICP estimation, although it must be validated in further studies before any considerations regarding its efficacy and accuracy for this purpose can be made (Table 1) [25]. Given the huge differences in the described methods for ONSD ultrasound estimation among all studies, there is undoubtedly a need for establishment of a formal protocol that considers the aforementioned parameters, includes details that may not be as obvious (such as the position of the patient’s gaze during measurement), and can be applied across different regions of the world. Currently, there are emerging data that could represent important starting points, such as anatomical landmark identification and a quality checklist [44, 45].

Overall, ONSD correlations with ICPi were moderate to high in the reviewed studies (Table 1), with all of them reporting ONSD values greater than 5 mm corresponding with elevated ICP values (> 20 mm Hg). However, the highest sensitivity, specificity, predictive ability for high ICP values [23] and diagnostic accuracy [18] were seen with values greater than or equal to 5.85 mm. This effect was even more pronounced for values exceeding 6 mm, in line with concomitant ICP increases. Specific data for different ICP thresholds were given by Launey et al. and Soliman et al. (Table 1) [20, 29]. In the near future, we need more studies that consider standardized ultrasound techniques for ONSD measurement based on criteria for quality, definitions of intracranial hypertension (based on physiology rather than simple ICP values), decompressive craniectomy-induced changes in ONSD estimations, ONSD correlations with ICP properties (e.g., waveform trends), and site-of-injury descriptions to help identify compartmentalized vs. diffuse ICP changes.

## Limitations

This review, as their first and third counterparts, has multiple limitations. First, only studies in patients 18 years or older were included, potentially leaving out valuable information from patients 16 years and older who are also considered part of the “adult population.” Second, as a matter of a scoping review design, in-depth statistical analyses or risk-of-bias assessments that are usually performed in systematic reviews were not done in our study. This may represent a weakness for data interpretation in terms of diagnostic accuracy and nICP-ICPi correlation comparison between studies. Third, studies that assessed other ICPi monitoring techniques (e.g., opening pressure by lumbar puncture) were not included in our review, given that intraventricular/intraparenchymal catheters are still considered the gold standard. This is a potential loss of valuable additional information. Notwithstanding, we agree that this paper can be seen as complementary to the aforementioned work by Aletreby et al. regarding ONSD for nICP monitoring [46]. Finally, we only included studies in patients with TBI. As such, the analyses and conclusions derived from this work cannot be extrapolated to other neurocritical care patient populations (e.g., aSAH, ischemic stroke, or intracerebral hemorrhage).

## Conclusions

This review can be considered an update acknowledging ONSD as possibly (when properly done), one of the most practical, fast, and reliable methods for nICP monitoring in patients with TBI. ONSD cutoffs that correlate with ICPi values were provided, although it must be underscored that these still cannot be considered a replacement for invasive techniques. ONSD seems to be promising for bedside identification of patients with high degrees of neurological deterioration secondary to intracranial hypertension.

### Supplementary Information

Below is the link to the electronic supplementary material.

Supplementary file 1 (DOCX 164 kb)
