# Supplementary figures and images for: Using Optic Nerve Sheath Diameter for Intracranial Pressure (ICP) Monitoring in Traumatic Brain Injury: A Scoping Review
Source: Neurocrit Care. 2023 Dec 19;40(3):1193–212. doi: 10.1007/s12028-023-01884-1 (PMC11147909; doi:10.1007/s12028-023-01884-1)

**Appendix 1.**

**EMBASE Search Strategy**

**
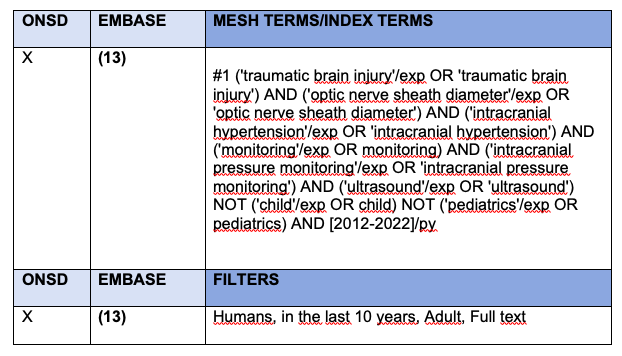
**

**Appendix 2.**

**PUBMED Search Strategy**

**
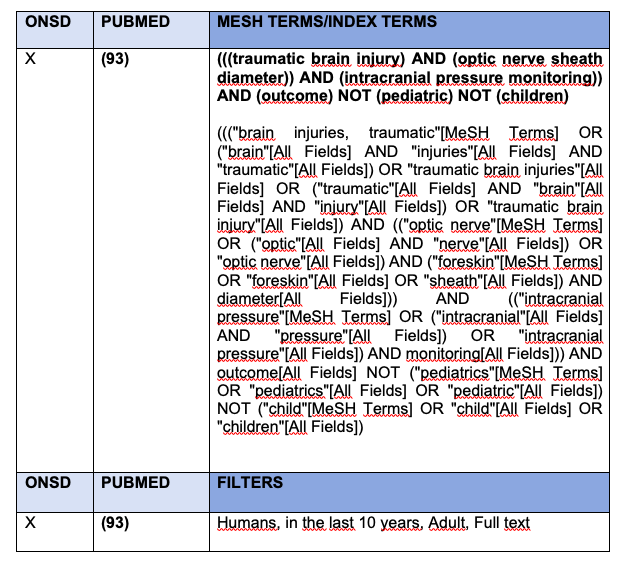
**

Supplement: Supplementary file 1 — (DOCX 164 kb) [file 12028_2023_1884_MOESM1_ESM.docx]
